# A high-density collection of EMS-induced mutations for TILLING in Landsberg *erecta *genetic background of *Arabidopsis*

**DOI:** 10.1186/1471-2229-9-147

**Published:** 2009-12-14

**Authors:** Beatriz Martín, Mercedes Ramiro, José M Martínez-Zapater, Carlos Alonso-Blanco

**Affiliations:** 1 Departamento de Genética Molecular de Plantas, Centro Nacional de Biotecnología (CNB), Consejo Superior de Investigaciones Científicas (CSIC), Madrid-28049, Spain

## Abstract

**Background:**

*Arabidopsis thaliana *is the main model species for plant molecular genetics studies and world-wide efforts are devoted to identify the function of all its genes. To this end, reverse genetics by TILLING (Targeting Induced Local Lesions IN Genomes) in a permanent collection of chemically induced mutants is providing a unique resource in Columbia genetic background. In this work, we aim to extend TILLING resources available in *A. thaliana *by developing a new population of ethyl methanesulphonate (EMS) induced mutants in the second commonest reference strain. In addition, we pursue to saturate the number of EMS induced mutations that can be tolerated by viable and fertile plants.

**Results:**

By mutagenizing with different EMS concentrations we have developed a permanent collection of 3712 M_2_/M_3 _independent mutant lines in the reference strain Landsberg *erecta *(L*er*) of *A. thaliana*. This population has been named as the Arabidopsis TILL*er *collection. The frequency of mutations per line was maximized by using M_1 _plants with low but sufficient seed fertility. Application of TILLING to search for mutants in 14 genes identified 21 to 46 mutations per gene, which correspond to a total of 450 mutations. Missense mutations were found for all genes while truncations were selected for all except one. We estimated that, on average, these lines carry one mutation every 89 kb, L*er *population providing a total of more than five million induced mutations. It is estimated that TILL*er *collection shows a two to three fold higher EMS mutation density per individual than previously reported *A. thaliana *population.

**Conclusions:**

Analysis of TILL*er *collection demonstrates its usefulness for large scale TILLING reverse genetics in another reference genetic background of *A. thaliana*. Comparisons with TILLING populations in other organisms indicate that this new *A. thaliana *collection carries the highest chemically induced mutation density per individual known in diploid species.

## Background

A major challenge in plant biology is the identification of biological functions for all genes from the main model plant species, *Arabidopsis thaliana *and rice. To this end, a large number of genetics and genomics resources are being developed in both model plants [1,2]. In particular, collections of induced mutants that can be screened by reverse genetics, such as T-DNA or transposon insertional mutants [3-5] provide a unique resource for functional studies. However, the mutational spectrum of insertional mutagenesis with effect on gene function is mostly limited to gene knock-out disruptions. Genes whose severe loss-of-function is lethal or highly pleiotropic cannot be functionally dissected with such mutants. In addition, the size of saturated populations containing insertion mutants randomly generated for most genes of an organism is extremely high because each line carries only a rather small number of mutations [6]. As a complementary resource, chemically induced mutants have been shown to provide an efficient alternative because each individual line can bear single point missense and nonsense substitutions in hundreds of genes [7]. Therefore, an allelic series of induced mutations with different effects on gene function can be easily isolated by screening a few thousands mutagenized plants [6].

In the past few years, chemically induced mutants have become a major resource for reverse genetics studies thanks to the development of TILLING (Targeting Induced Local Lesions IN Genomes) [8]. TILLING enables the reverse selection of single point mutations by cleavage of mismatches in heteroduplex DNA with the endonuclease CEL I. This powerful strategy was first applied in an *A. thaliana *mutant collection induced with ethyl methanesulphonate (EMS) [9,10] in the commonest genetic background Columbia (Col) whose genome sequence had been first completed [11]. Since then, TILLING collections of EMS induced mutants have been developed in a large number of plant species including rice, maize, barley, sorghum, wheat, *Brassica napus*, *B. oleracea *and *Medicago truncatula*, as well as model animals like *Drosophila *and *Caenorhabditis elegans *[12-21]. In most of these EMS mutant collections, reference genetic backgrounds of wide and general interest are used. However, given the limitations of having mutations in a single genetic background, new populations of chemically induced mutants for TILLING analyses are currently being developed in other reference strains of several species like rice or soybean [22,23]. In addition, the quality of TILLING mutant populations is determined by the density of mutations per individual, since this limits the size of allelic series than can be isolated for each gene and the size of a saturated genome population. For this reason, other TILLING populations have been developed in rice, barley, soybean or *M. truncatula*, aiming to increase the amount of mutations per line by either using different mutagens like sodium azide and N-methyl-N-nitrosourea or increasing the mutagen dose [12,22-25].

In *A. thaliana*, several reference genetic backgrounds are widely used such as Col or Landsberg *erecta *(L*er*). The latter is the second most commonly studied strain because many mutants have been classically isolated in it and a large portion of its genome sequence was available soon after Col sequence [26]. In this work we have developed a new collection of *A. thaliana *EMS induced mutants for TILLING reverse genetics, aiming at two major objectives. First, to extend TILLING resources in *A. thaliana *by using L*er *reference genetic background, for which reverse genetic tools are rather limited. Second, to enrich the number of independent mutations available in this collection as much as possible by increasing the density of mutations per line. TILLING evaluation of this population for several gene fragments indicates that it carries the largest density of chemically induced mutations reported in diploid organisms, hence demonstrating its usefulness for reverse selection of mutants.

## Results

### Generation of a permanent collection of highly EMS-mutagenized lines in *Arabidopsis*

**Figure 1:**
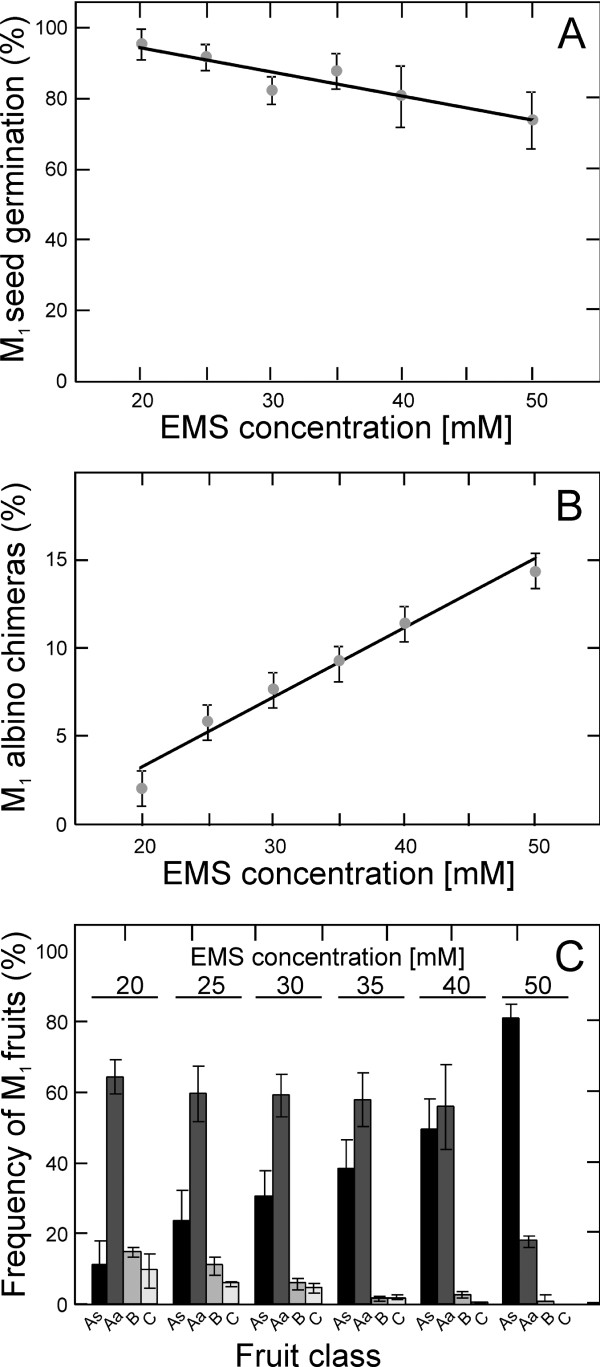
**Dose effects of EMS mutagenesis in *A. thaliana *M_1 _L*er *plants**. A) Frequency of M_1 _seed germination. B) Frequency of M_1 _albino chimeras at vegetative stage. C) Frequency of sterility, embryo lethality and fertility measured as the percentage of different fruit classes. Fruits of M_1 _plants are classified as As, Aa, B and C (from fully sterile to nearly normal fertility) depending on the proportion of aborted seeds (see Methods for details). Data are mean ± SE of three to six replicates. Untreated control plants showed 100% germination, 0% albino chimeras and 0, 3 and 97% of A, B and C fruit classes.

To obtain a new population of chemically induced mutant lines useful for reverse genetic studies in *Arabidopsis thaliana*, seeds of the Landsberg *erecta *(L*er*) *glabrous1-1 *genotype were mutagenized with EMS at concentrations of 20 to 50 mM (Figure 1). The effects of EMS and the efficiency of the mutagenesis treatment were estimated by quantifying three parameters on M_1 _plants: seed germination, frequency of albino chimeras and fertility (see Methods). Germination of M_1 _seeds was negatively correlated with EMS dose (*r *= -0.93; *p *= 0.008), while the frequency of M_1 _albino chimeras increased with concentration (*r *= 0.99; *p *= 0.001) (Figure 1A and 1B). Seed fertility of M_1 _plants and the degree of M_2 _embryo lethality was quantified by estimating the proportion of fully or nearly sterile fruits (classes As and Aa) and the proportion of semi or normal fertile fruits (classes B and C). As shown in Figure 1C, the total frequency of class A fruits increased linearly with EMS concentration, whereas the frequency of fertile fruits rapidly decreased. To maximize the frequency of mutations per individual, only M_1 _plants from treatments showing a frequency of fertile fruits smaller than 35% but larger than 2% were individually harvested. A total of 3712 M_2 _families were grown to isolate individual M_2 _DNA and to harvest their M_3 _offspring seeds. To ensure independence of the mutations present in this population, a single M_2 _plant was harvested from each M_1 _plant. In agreement with the high proportion of embryo lethality, all M_2 _families segregated for easily visible morphological mutations (data not shown). Fifty six percent of M_2 _lines were derived from 25 mM EMS mutagenesis, and on average, EMS treatments used to obtain the collection show less than 25% fertile fruits (Table 1). The DNA of M_2 _plants and the M_3 _seeds of the 3712 lines were stored (see Methods) providing a permanent population of mutant lines for TILLING analysis in L*er *genetic background. This population has been named as the Arabidopsis TILL*er *collection.

**Table 1:** Description of *A. thaliana *L*er *mutant lines and mutations in relation to EMS dose.

EMS dose (mM)	Number of TILL*er * lines	Mean (B+C) fertility class (%)	Total number of screened lines*	Number of mutations	Het/Hom mutation ratio	Number of lines with two mutations	Density of mutations per line
20	46	24.5 ± 5.4	614 (1.5%)	7 (1.5%)	6.0	0 (0%)	1/84 kb
25	2082	16.8 ± 3.7	21150 (50.8%)	178 (39.6%)	3.6	10 (29.4%)	1/114 kb
30	741	10.5 ± 2.8	9196 (22.1%)	112 (24.9%)	3.3	10 (29.4%)	1/79 kb
35	739	3.7 ± 1.2	9313 (22.4%)	128 (28.4%)	4.3	12 (35.3%)	1/70 kb
40	104	2.6 ± 1.0	1335 (3.2%)	25 (5.6%)	4.0	2 (5.9%)	1/51 kb
**Total**	3712	12.6 ± 4.6**	41608	450	3.7**	34	1/96 kb**

*: Number of screened lines is estimated as the total sum of individuals analyzed for each of the 14 gene fragments.

**: Weighted averages are shown according to frequency of lines of the different EMS dose.

### Mutation frequency, distribution and functional spectrum in TILL*er *collection

**Figure 2:**
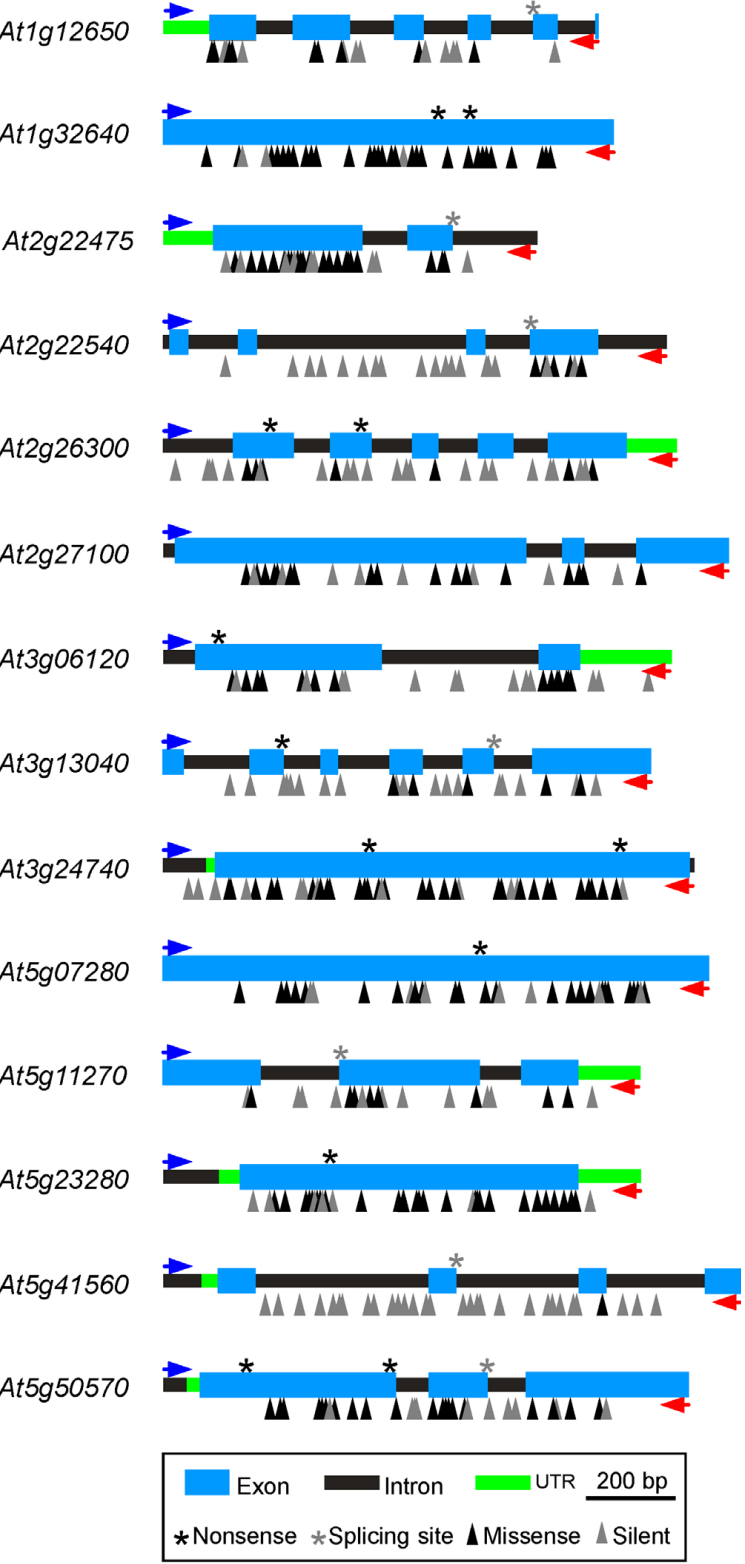
**Gene fragments analyzed and mutations found in TILL*er *collection**. Positions of silent, missense, splicing site and nonsense mutations are indicated by grey arrow heads, black arrow heads, grey asterisks and black asterisks, respectively.

The quality of this collection for mutant discovery was evaluated after the analysis of 14 gene fragments distributed among four of the five *A. thaliana *chromosomes and chosen from requests by different research laboratories (see Methods; Figure 2 and Table 2). These fragments have a GC content similar to that of fragments studied in Col background TILLING collection [10] and to average coding regions of *A. thaliana *genome [11]. On average, amplicons were 1.1 kb long and 62.2% corresponded to exon sequence, which is similar to genome average exon proportion [11]. In total, we found and confirmed by sequencing 450 mutations in the 15.7 kb analyzed from different amplicons. All mutations corresponded to G/C to A/T transitions, in agreement with the nearly unique type of nucleotide substitutions observed in previous analyses of EMS-mutagenized *A. thaliana *plants [10]. The distribution of mutations among the EMS doses of the lines was independent of the number of lines per dose (Table 1; χ^2 ^= 28.9; df = 4; *p *< 0.0001). A larger number of mutations were found at 30, 35 and 40 mM than expected from the number of lines, while the opposite was found at 25 mM. In addition, 34 lines carried several mutations in the same or two different gene fragments (Table 1). An excess of these lines was found in plants derived from high EMS concentrations (≥30 mM) while a deficiency appears in low EMS concentrations (≤25 mM) when comparing with the expected number according to the proportion of lines from each EMS dose (χ^2 ^= 5.8; df = 1; *p *= 0.016). Therefore, the higher the EMS concentration used to obtain the lines, the larger the number of mutations per line.

**Table 2:** Mutations found in 14 gene fragments analyzed in TILL*er *collection.

Gene	Amplicon length (bp)	Exon length (bp)	Intron length (bp)	GC content (%)	# of screened lines	Total # of mutations	Mutation class*		
							
							Silent	Missense	Truncation**
At1g12650	977	416	561	35.6	1968	22	11/1	6/3	1/0
At1g32640	1015	1015	0	49.8	2000	42	8/2	22/7	3/0
At2g22475	839	439	400	44.3	3416	33	7/2	16/7	1/0
At2g22540	1128	278	850	35.5	3216	25	18/2	4/0	1/0
At2g26300	1152	547	605	37.7	3456	32	19/3	6/2	2/0
At2g27100	1272	1049	223	43.8	3360	25	6/2	14/3	0/0
At3g06120	1143	513	630	35.0	3432	29	11/3	10/4	1/0
At3g13040	1093	581	512	39.8	2728	28	17/3	4/2	2/0
At3g24740	1195	1068	127	46.4	3472	46	10/3	23/8	2/0
At5g07280	1228	1228	0	46.0	2728	36	6/2	17/10	1/0
At5g11270	1075	667	408	39.8	1896	21	7/5	4/4	1/0
At5g23280	1077	762	315	42.5	3352	39	7/1	23/7	1/0
At5g41560	1299	262	1037	36.8	3200	32	25/5	0/1	1/0
At5g50570	1180	939	241	40.3	3384	40	12/1	23/1	2/1

**Total**	15673	9764	5909	41.0	41608	450	164/35	172/59	19/1
**Average**	1120	697	422	41.0	2972	32	12/3	12/4	1/0

**Observed frequency (%)**							**47.2**	**48.5**	**4.3**
**Expected frequency (%)**							**48.8**	**46.1**	**5.1**

*: number of heterozygous/homozygous mutations are given for each of the three mutations classes.

**: Truncations include mutations generating premature stop codons and mutations in splice sites.

On average we analyzed 2972 lines per fragment and detected 10.8 mutations per 1000 mutant lines (Table 2). Twenty-one to 46 mutations were found per fragment, and in most gene fragments there was a reduction of mutation detection in the ~100 bp terminal segments (Figure 2), as expected from LI-COR detection system (see Methods). However, mutations appeared evenly distributed along the rest of the gene fragments within exon and intron regions (Figure 2).

For all but one gene fragment we found mutations of three classes according to their predicted effects on protein structure: silent, missense and truncation mutations (Table 2). The observed frequencies of the three classes of mutations fitted the expected frequencies of silent, missense and truncations, respectively, as estimated by CODDLE analyses (χ^2 ^= 1.7; df = 2; *p *= 0.42). Truncations include nonsense mutations generating premature stop codons and mutations in intron splice sites, the observed frequencies of both classes (2.5% and 1.8% respectively) also fitting expected frequencies (4.0% and 1.1%)(χ^2^ = 2.8; df = 1; *p *= 0.09). Interestingly, truncation mutations were obtained for 13 of the 14 fragments, as expected from their 5.1% frequency and the large average number of mutations found per gene (1- [1-0.05]^32 ^= 0.81 probability).

As shown in Table 2, an average ratio of heterozygous/homozygous mutations of 3.7 was found, which is significantly different from the expected 2:1 proportion for M_2 _plants (χ^2 ^= 30.2; df = 1; *p *< 0.0001). Although an excess of heterozygotes appeared for silent mutations (*p *< 0.01), this ratio was extreme for truncations since all but one of such mutations were present as heterozygotes. In addition, distortion from the expected proportion was larger for high EMS dose lines (35-40 mM) than for low concentrations (25-30 mM) (Table 1).

From these analyses we estimated an average density of detected mutations per line of 1 mutation per 89 kb (450 mutations/[2972 lines × 13.4 kb]), which was calculated after subtracting 160 terminal base pairs with low LI-COR detection, from each amplicon (see Methods). However, the density of mutations varied from 1/114 kb to 1/51 kb depending on the EMS dose used to generate the lines, a two-fold variation being found between 25 and 40 mM (Table 1). To contrast this average mutation frequency estimation, the density of mutations was also independently calculated from the number of pool samples with two mutant individuals in the same fragment or from the number of individual lines with two mutations in the same gene fragment [10]. Forty-four pool samples were found to carry two mutant individuals when analyzing the individual lines. Thus, a total of 406 pool samples were originally detected as positive pools, which contain 406 × 8 individuals representing a sample analyzed at individual level to find second mutations. From these 406 pool samples with at least one positive line we estimated a density of 1 mutation/71 kb (44/[406 pool samples × 8 individuals × 0.96 kb]), which is similar to previous estimate. On the other hand, when sequencing positive lines for their corresponding fragments, five individual lines were found to carry two mutations within the same fragment. Therefore, 445 lines were sequenced and can be considered a sample analyzed to detect second mutations by sequencing. From these lines we calculated a density of 1 mutation/100 kb (5/[445 × 1.12 kb]), which is comparable to the above estimates. In contrast to previous calculations, this latest density was estimated from the complete amplicon length (1.12 kb) because it was derived from the sequencing of entire fragments and not from LI-COR detection of positive lines.

From the above density of mutations we have calculated an average number of 1404 mutations per line, the complete TILL*er *collection providing a total of 5.2 million mutations. Taking into account the observed frequencies of truncation and missense mutations (Table 2), and the total length of gene regions of *A. thaliana *genome (see Methods), we have roughly estimated that each TILL*er *line contains, on average, 30 genes with knock-out mutations and 281 genes with aminoacid substitutions.

## Discussion

We have developed a new permanent collection of 3712 independent EMS-induced mutant lines for reverse genetic analysis in the reference laboratory strain Landsberg *erecta *of *A. thaliana*. To maximize the number of mutations present in this population we have increased the frequency of mutations per M_2_/M_3 _line by using M_1 _plants with lower seed fertility than that of plants used to obtain the existing population in Columbia background [9]. By compromising fertility, we aimed to saturate the number of chemically induced mutations that can be tolerated by *A. thaliana *plants that are still viable and able of sexual reproduction. We estimated that, on average, the lines of this new L*er *collection carry one mutation every 89 kb, which is significantly larger than the density of 1/300 kb estimated in current Col population [10]. As expected, we found that the higher the EMS concentration the higher the density of detected mutations per line. Thus, experimental control of EMS mutagenesis enables substantial increase of the frequency of induced mutations in viable and seed fertile plants. However, we cannot discard that mutation density differences between both TILLING populations of *A. thaliana *might be partly due to natural genetic variation between both wild type strains for their tolerance to chemically induced mutations. Accordingly, it could be speculated that such natural variation might be determined by variation for reproductive system plasticity or for DNA repair mechanisms.

As described by Greene *et al. *[10] estimations of the density of chemically induced mutations detected by TILLING procedure can be biased due to several factors such as: 1) uneven mutation detection among the pools of eight plants; 2) uneven mutation detection along the length of gene fragments; 3) higher GC content of analyzed fragments (41%) than average genome (35%) [11]; and 4) dilution of one fourth of the M_1 _mutations in M_2 _plants. Taking into account these factors, it has been estimated that Col population shows a corrected average mutation density per line of 1/170 kb. Despite these factors, the 1/89 kb average frequency of induced mutations detected in L*er *population is nearly twice higher than that of Col. Furthermore, L*er *lines obtained from the highest EMS dose show a 1/51 kb mutation density, which triplicates the lowest density estimate of Col population. Comparisons with TILLING populations in other species indicates that *A. thaliana *L*er *collection carries the highest mutation density induced by different chemical agents in diploid plants, including *Brassica oleracea*, *Medicago truncatula*, rice, barley, maize and sorghum; or animals like *Drosophila *and *Caenorhabditis elegans*, or the oomycote *Phytophthora sojae *(see Table 3). Only rapeseed and wheat collections carry a higher density of mutations, as expected from their polyploid nature and consequently, their higher tolerance to loss-of-function mutations due to gene duplications and redundancies (Table 3). Thus, the density of mutations found in L*er A. thaliana *population increases the estimated load of chemically induced mutations that diploid species can tolerate in sexually fertile individuals.

**Table 3:** Frequency of chemically induced mutations reported by TILLING in different species.

Species	Ploidy level	Mutagen	Mutation density per line	Reference
*A. thaliana *(Landsberg *erecta*)	2×	EMS	1/89 kb	*present work*
*A. thaliana *(Columbia)	2×	EMS	1/300 kb	[10]
*Brassica oleracea*	2×	EMS	1/447 kb	[18]
*Brassica napus*	4×	EMS	1/41.5 kb	[17]
*Medicago truncatula*	2×	EMS	1/400 kb	[19]
*Medicago truncatula*	2×	EMS	1/485 kb	[25]
*Glycine max*	4×	EMS	1/140 to 1/550 kb	[23]
*Glycine max*	4×	NMU	1/140 kb	[23]
*Oryza sativa*	2×	EMS	1/294 kb	[12]
*Oryza sativa*	2×	NaN_3 _+ MNU	1/265 kb	[12]
*Oryza sativa*	2×	MNU	1/135 kb	[22]
*Zea mays*	2×	EMS	1/485 kb	[13]
*Sorghum bicolor*	2×	EMS	1/526 kb	[15]
*Hordeum vulgare*	2×	EMS	1/1000 kb	[14]
*Hordeum vulgare*	2×	NaN_3_	1/374 kb	[24]
*Triticum aestivum*	6×	EMS	1/40 kb	[16]
*Triticum durum*	4×	EMS	1/25 kb	[16]
*Caenorhabditis elegans*	2×	EMS	1/293 kb	[21]
*Drosophila melanogaster*	2×	EMS	1/91 to 1/156 kb	[20]
*Phytophthora sojae*	2×	ENU	1/142 kb	[30]

NaN_3_: sodium azide; MNU: N-methyl-N-nitrosourea; ENU: ethylnitrosourea.

The two *A. thaliana *TILLING populations, L*er *and Col, also differ in the proportion of heterozygous:homozygous mutations recovered in TILLING analyses, L*er *showing substantially higher total average ratio than Col (3.7 *versus *2.1, respectively) [10]. The largest deficiency of homozygous mutations corresponds to truncations, which shows the largest difference between both populations (ratio of 3.7 *vs*. 19 for Col and L*er*, respectively). Therefore, a stronger negative selection against deleterious mutations seems to affect L*er *than Col collection. This is probably a consequence of the extreme high-density of mutations present in L*er *lines, since the maximum load of deleterious induced mutations that can be tolerated by a viable and fertile M_2 _plant will likely be determined by a threshold number of homozygous truncations and deleterious missense mutations. M_2 _plants carrying a higher number of homozygous deleterious mutations than this threshold will not be viable or fertile. Given the self-fertilizing nature of *A. thaliana*, the higher the M_1 _mutation density, the higher the proportion of M_2 _offspring plants that will surpass the maximum number of homozygous deleterious mutations and, consequently, stronger selection against such mutations. Thus, higher M_1 _mutation densities will lead to higher M_2 _ratios of heterozygous/homozygous mutations due to lower frequency of M_2 _plants below the threshold of homozygous deleterious mutations. This relationship is supported by the larger ratios observed in L*er *lines with high mutation density generated with EMS doses ≥35 mM, than in lines with lower density obtained with 25-30 mM EMS. Nevertheless, presumed silent mutations including synonymous and intronic mutations also showed a significant defect of homozygotes in L*er *collection, whereas this was not observed for missense mutations. Potential genetic mechanisms accounting for this unexpected result are unknown but it cannot be discarded that the genes surveyed in this work are biased for the deleterious effect of their mutations. In agreement, other *A. thaliana *public mutant collections do not contain mutations in several of the genes analyzed here http://www.arabidopsis.org suggesting that mutations in their coding and non-coding regions show stronger deleterious defects than genome average.

## Conclusions

The TILL*er *collection generated in this work provides a new resource for reverse selection of EMS induced mutants in *A. thaliana*. The high mutation density of this population increases the size of allelic series that can be obtained and reduces the population size that needs to be screened. However, this high mutation density implies that more backcrosses are required to eliminate undesired background mutations in selected mutant lines. It has been estimated that 20 mutations are necessary to have 0.95 probability of finding a missense deleterious mutation per gene [6]. Considering the ~50% observed frequency of missense mutations and assuming that 25% of them are deleterious, we have calculated that on average, 1774 TILL*er *lines are sufficient to obtain 20 mutations per ~1 kb gene fragment, while the larger analyses carried out until now are providing additional truncation mutations for ~90% of the genes. Currently, TILL*er *collection is screened as a public service to search for mutants in genes of interest for any laboratory http://www.cnb.csic.es/~tiller/. The availability of another TILLING service in the second commonest reference genetic background of *A. thaliana *enables deeper gene functional studies such as those aiming to uncover new gene effects or interactions of specific mutations with genetic backgrounds. Given the success of current existing collections, it can be expected that the use of chemically induced genetic variation will further extend in the near future with the development of similar resources in other reference strains and/or using other mutagens.

## Methods

### Mutagenesis

Seeds of the laboratory strain Landsberg *erecta *carrying the marker mutation *glabrous1-1 *were mutageniced with ethyl methanesulphonate (EMS) [9]. Fresh M_0 _seeds were treated with 20, 25, 30, 35, 40 or 50 mM EMS during 17 hours in 10 ml vials containing 2500 seeds. Three to eight batches of 2500 seeds (vials) were treated at each dose. After thorough washing, M_1 _seeds were sown on pots with soil:vermiculite mix at 3:1 proportion, in a 20°C greenhouse supplemented with lamps to provide a 16 hours light:8 hours darkness photoperiod. To estimate the EMS effects and the quality of the mutagenesis we quantified germination of M_1 _seeds, and the proportion of chimeric M_1 _plants that show albino or yellow sectors at the vegetative stage of six-eight leaves (albino chimeras). In addition, seed fertility and degree of embryo lethality of the M_1 _plants were estimated as previously described [9] with the following modifications. For each EMS dose, 10 mature siliques of the main inflorescence from 10 M_1 _plants were dissected under a stereomicroscope and the number of normal and aborted seeds was counted. From these data, fruits were classified in four classes according to their proportion of normal M_2 _seeds and M_2 _defective embryos. Class As is completely sterile and has no seed, either normal or aborted; class Aa has a 3:1 proportion of normal:aborted seeds, or smaller (aborted seeds >20%); class B shows 4:1 to 13:1 proportions (20% ≥ aborted seeds >6.7%); and class C has 14:1 or larger proportion (nearly normal fertile fruits with less than 6.7% aborted seeds). M_1 _plants were individually harvested and treatments with a frequency of fertile fruits (B+C) larger than 2% or smaller than 35% were used to generate the M_2_/M_3 _lines that are part of TILL*er *collection (Table 1). Mutageniced batches with more than 35% or less than 2% fertile fruits were discarded independently of the concentration of their EMS dose.

### Development of mutant and DNA collections

Five to sixteen M_2 _offspring seedlings were grown from each M_1 _individual and tissue was collected from a single M_2 _fertile plant. M_3 _seeds of each M_2 _selected plant were individually harvested and stored. To ensure enough tissue and M_3 _offspring seeds from a single M_2 _plant, each family was grown on a 0.9 l. pot that was maintained until fruit formation in a growth chamber illuminated with cool-white fluorescent lamps that provide a short day photoperiod of 8 hours light:16 hours darkness. DNA was isolated as previously described [27] without mercaptoethanol. The DNA of 3712 M_2 _plants was quantified, diluted and arrayed in a total of 58 (8 × 8)-individual plates as described in [28]. The DNA was combined in groups of eight individuals using a one-dimension pooling strategy. Thus, the 3712 samples of the collection were arranged in five (96 × 8)-pool plates, four containing the DNA of 768 individuals and one from 640 individuals.

### Mutation detection

Mutations in gene fragments were detected using the TILLING procedure developed and described by Till *et al*. [9,28]. Briefly, primers for amplification of target genes were designed using the CODDLE and Primer 3 system http://blocks.fhcrc.org/proweb/input/. Forward and reverse primers were labeled with IRDye 700 and IRDye 800 respectively. PCR, heteroduplex DNA formation and heteroduplex digestion with CEL I were carried out using the 768 pool plates containing 5 μl of DNA at 0.15 ηg/μl as previously described [28]. CEL I was purified from celery juice extracts according to [29] with minor modifications. For that, concentrated extracts were incubated with concanavalin A-sepharose, followed by chromatography purification steps with DEAE FF and Q columns using an AKTA FPLC system (Amersham). Cleaved DNA fragments were separated in a LI-COR 4300 DNA analyzer and gel images were manually analyzed using Photoshop (Adobe system) to find positive pools. Thereafter, individual plates containing the eight individual DNA samples of each positive pool were similarly analyzed after mixing with control wild type DNA. Validated mutants were sequenced and sequences were analyzed with Chromas and DNASTAR softwares. Mutation frequencies were calculated as described in [10] by subtracting 160 bp from the size of each amplicon due to the observed low ability of LI-COR system for mutation detection in the 80 bp terminal segments of gene fragments. To enable direct comparisons of mutation frequencies among TILLING projects from different species [10,12-25,30], these calculations were based on haploid sizes of the analyzed fragments. Thus, frequencies are given per kb of diploid genome and should be divided by two when taking into account the diploid nature of *A. thaliana*. The total number of mutations was calculated using an *A. thaliana *genome size of 125 Mb and a total size of gene regions of 33249 kb (exons) plus 18055 kb (introns) [11].

## Authors' contributions

BM participated in the generation of the mutant collection, in the analysis of mutations and in the writing of the manuscript. MR participated in the generation of the mutant collection and in the analysis of mutations. JMZ participated in the conception and design of the study and in the writing of manuscript. CAB participated in the conception, design and supervision of the study, in the analysis of mutations and in the writing of manuscript. All authors read and approved the final manuscript.
